# Prior episode of colitis impairs contextual fear memory

**DOI:** 10.1186/s13041-022-00961-4

**Published:** 2022-08-29

**Authors:** Chia-Shan Wu, Valerie Endres

**Affiliations:** Department of Nutrition, Texas A&M University, 123 Cater-Mattil, 2253 TAMU, College Station, TX 77843 USA

**Keywords:** Inflammatory bowel disease, Ulcerative colitis, Conditioned fear, Contextual fear memory

## Abstract

Accumulating evidence has shown that intestinal inflammations in inflammatory bowel disease (IBD) also drive pathological responses in organs outside the intestine, including the brain. Previous studies using the dextran sodium sulfate (DSS)-induced colitis model have shown that colonic inflammation contributes to the development of anxiety- and depression-related behaviors; however, little is known about whether memory function is affected. Here, we subjected male and female C57BL/6J mice to DSS-induced colitis for 6 days, followed by Pavlovian conditioned fear (CF) tests 15 days after the start of inflammation, when local colonic inflammation has receded. The contextual and cued CF tests were used to assess associative fear memory. We found that DSS-induced colitis led to significant impairment in contextual fear memory in both male and female mice; on the other hand, auditory cued fear memories were comparable between control and DSS-treated mice. There were marked signs of astrogliosis in the hippocampal regions 17 days (D17) after colitis induction. Furthermore, molecular characterization of hippocampi showed marked but transient increases in the expression of inflammatory genes *Nfkb*, *Trem2* (microglial marker), *GFAP* (astrocyte marker), *Il1b*, and *S100a8* in DSS-treated mice. While the expression of *Nfkb*, *Trem2*, and *GFAP* showed a peak on day 10, the *S100a8* expression was high on days 10 and 17 and subsided on day 42. Interestingly, expression of *Bdnf* remained elevated in the times assessed (D10, 17, 42). Together, these results demonstrated that DSS-induced colitis could induce prolonged neuroinflammation and impaired contextual fear memory.

**Supplementary Information:**

The online version contains supplementary material available at 10.1186/s13041-022-00961-4.

## Main text

The prevalence of inflammatory bowel disease (IBD), a chronic inflammatory condition of the gastrointestinal tract, continues to rise [1]. IBDs, including Crohn’s disease and ulcerative colitis, are chronic conditions that cycle between periods of active flare and remission. In addition to primary pathologies affecting the intestine, IBD has been linked to neuroinflammation and affects emotional functions including depression and anxiety [2].

To model IBD in rodents, dextran sulfate sodium (DSS)-induced colitis has been widely used, which elicits intestinal pathologies similar to human ulcerative colitis [3]. Previous studies have shown that DSS-induced colon inflammation led to increased brain excitability and neuroinflammatory phenotype, including the transcriptional increase of pro-inflammatory genes, and infiltration of monocytes and neutrophils [4–6]. While DSS-induced colitis has been shown to affect stress-related behaviors and increase anxiety- and depression-like behaviors [5–8], little is known about whether a prior episode of colitis affects memory function.

**Fig. 1:**
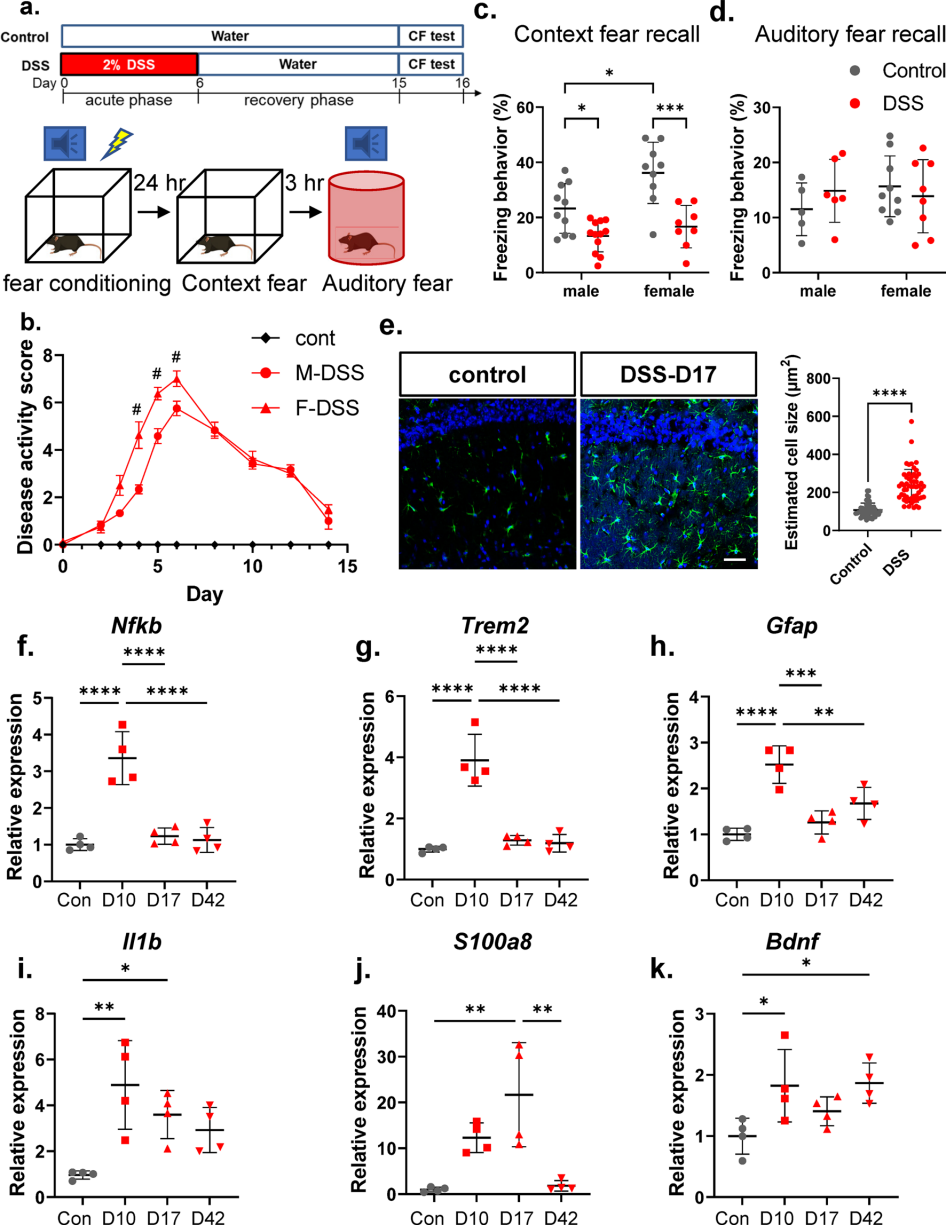
Prior exposure to DSS-induced colitis led to neuroinflammation. **a** Schematic diagram of experimental design. Mice were given normal (control) or 2% DSS in drinking water for 6 days (Day 0–6), then switched to normal drinking water and allowed to recover, mimicking clinical remission. Mice were then subjected to conditioned fear (CF) tests on days 15–16. For in vivo study, n = 10, 12, 9, and 8 for male-control, male-DSS, female-control, and female-DSS groups, respectively. **b** Disease activities including fecal consistency and rectal pathologies were monitored. Data were analyzed with two-way ANOVA (treatment × repeated measures) followed by Tukey’s multiple comparisons test. ^#^p < 0.05 male-DSS vs. female-DSS. **c**, **d** DSS-exposed mice showed significantly impaired contextual fear memory in both male and female mice (**c**), but comparable auditory fear memory (**d**). Two-way ANOVA (treatment and gender as independent factors) followed by Tukey’s multiple comparisons test. **e** Representative images of the hippocampal regions of mice on Day 17. DSS-exposed mice showed increased astrogliosis (increase in abundance and cell size of GFAP-labeled astrocytes (green). Sections were counterstained with DAPI (blue). Scale bar 50 μm. Images were taken from 3 stained sections per brain, 3 brains per group. Images were processed and the areas of GFAP-labeled cells were quantified with ImageJ. Quantitative PCR analyses of expression of *Nfkb*, *Trem-2*, *Gfap*, *IL-1b*, *S100a8*, and *Bdnf* in the hippocampus collected from control and DSS-treated mice on Day 10, 17, and 42 (**f**–**k** respectively; n = 4 per group). One-way ANOVA followed by Tukey’s multiple comparisons test, *P < 0.05, **P < 0.01, ***P < 0.001, and ****P < 0.0001. All data were presented as mean ± SD, except for 1b where disease scores were presented as mean ± SEM, for clarity

In the present study, we subjected male and female C57BL/6J mice to DSS-induced colitis for 6 days, followed by Pavlovian conditioned fear (CF) tests 15 days after the start of colitis induction (Fig. 1a), when local colonic inflammation had receded. The contextual and cued fear conditioning tests are widely used to evaluate associative fear learning and memory [9]. Given the importance of the gut microbiome in contributing to the pathogenesis of ulcerative colitis, experimental mice were maintained on semi-purified OpenSource diets D12450J (Research Diets Inc.) to ensure consistency and reproducibility of the induced disease course. Mice received normal drinking water (control group) or DSS (2%) (MP Biomedicals; 36–50 kDa) in drinking water for 6 consecutive days to induce acute colitis, then switched back to normal drinking water. All mice were assessed for body weight, fecal consistency, and macroscopic fecal blood scores; detailed methods are described in Additional file 1. Experimental procedures were approved by the animal care committee of Texas A&M University.

Mice given 2% DSS for 6 days exhibited significant disease activities (composite score of fecal consistency and macroscopic fecal blood scores) and recovered gradually after cessation of DSS (Fig. 1b). Female C57BL/6J mice developed more severe disease symptoms at days 4–6 compared to male mice, but recovered to similar levels after cessation of DSS. On training day, mice received a mild foot shock conditioned with tone (Fig. 1a). 24 h later, mice were tested for contextual fear recall and placed in the same chamber for 5 min. Freezing behavior is used as an index of fear memory recall. We found that a prior episode of DSS-induced colitis significantly reduced contextual fear memory in both male and female mice, even when colitis-associated disease symptoms such as diarrhea and rectal bleeding have subsided (treatment: F_1, 35_ = 29.1, p < 0.0001, gender: F_1, 35_ = 8.939, p = 0.0051, treatment × gender: F_1, 35_ = 3.015, p = 0.0913) (Fig. 1c). In the auditory cue test, mice were placed into a novel-shaped chamber with altered coloring, flooring and odor, and the freezing behavior in response to the tone was assessed (percent freezing during the tone presentation *minus* percent freezing in the pre-stimulus phase). Some male mice showed cued fear response below the 5% threshold and were excluded from data analysis, while all female mice responded. Overall, freezing behaviors for auditory cues were comparable between control and DSS-treated mice (Fig. 1d).

It has been shown that contextual information is encoded by neurons in the hippocampus and conveyed directly to the amygdala, which generates conditioned fear responses [10, 11]. Next, we assessed astrogliosis as an indication of neuroinflammation [12], performing immunostaining of the astroglial protein GFAP (glial fibrillary acidic protein) on brain tissues collected on day 17. Astrogliosis was determined by increased GFAP expression with hypertrophic morphology (enlarged cytoplasmic area and thickness of processes) [12]. We found increased GFAP-positive cells with hypertrophic morphology, resembling reactive astrocytes, in the hippocampus of DSS-treated mice (Fig. 1e). To further explore the temporal changes of the neuroinflammatory response to colonic inflammation, we collected hippocampi from control and DSS-treated mice at day 10, 17 and 42 after the colitis induction. Quantitative PCR data demonstrated that expression of inflammatory genes *Nfkb*, *Trem2* (microglial marker), *Gfap*, and *Il1b* were significantly increased on day 10 and decreased thereafter to basal levels by day 42, except for *Il1b* (Fig. 1f–i, respectively). Similarly, a previous study has demonstrated persistent elevation of *Il1b* mRNA in the hippocampus 4-weeks after acute colitis [6]. Interestingly, expression of the S100 calcium-binding proteins S100A8 (*S100a8*) peaked on day 17 and subsided to basal level on day 42 (Fig. 1j). S100A8 is a ligand for RAGE (receptor for advanced glycation end product); it has been reported that S100A8 accumulates in the brain before the appearance of Aβ plaques in mice overexpressing the precursor of Aβ [13]. Furthermore, the expression level of the neurotrophic factor *Bdnf* remained elevated compared to control (Fig. 1k), suggesting an ongoing repairing process in the hippocampus long after the resolution of clinical symptoms of colon inflammation.

In conclusion, our study showed that contextual memory function was negatively impacted by an episode of colitis, with prolonged neuroinflammation in the hippocampal regions. Further work is required to determine mechanistically the interactions between innate neuroinflammatory response and neurons encoding specific inputs to hippocampal-amygdala neurocircuit to affect contextual fear memory [11]. Of note, clinical functional MRI data showed that patients with active-stage ulcerative colitis exhibited decreased hippocampal/parahippocampal activity that correlated with memory loss [14]. Overall, our data suggest that in addition to clinical management of the symptoms of IBD, other strategies to monitor and reduce neuroinflammation may need to be considered to prevent potential progression to chronic disease conditions such as dementia or neurodegenerative diseases, given that IBD patients are at increased risk for neurodegenerative diseases including Parkinson’s disease and dementia [15].

## Supplementary Information

**Additional file 1.** Experimental Methods.

## Data Availability

The detailed methods are described in the Additional file 1. All data and materials are available from the corresponding author upon request.

## Abbreviations

*Bdnf*: Brain-derived neurotrophic factor
CF: Conditioned fear
DSS: Dextran sulfate sodium
GFAP: Glial fibrillary acidic protein
IBD: Inflammatory bowel disease
*Il1b*: Interleukin 1 beta
*Nfkb*: Nuclear factor kappa B
RAGE: Receptor for advanced glycation end product
*S100a8*: S100 calcium-binding proteins A8
*Trem2*: Triggering receptor expressed on myeloid cells 2
