# Supplementary material for: Prior episode of colitis impairs contextual fear memory
Source: Mol Brain. 2022 Aug 29;15:74. doi: 10.1186/s13041-022-00961-4 (PMC9426209; doi:10.1186/s13041-022-00961-4)
Supplement: Supplementary file 1 — Additional file 1. Experimental Methods. [file 13041_2022_961_MOESM1_ESM.pdf]

Additional File 1. Experimental Methods.

Animals

Male and female C57BL/6J mice at 3-4 months old were used in the study. Animals were born and raised in-house and maintained in groups of 3-5 under controlled temperature and lighting (22 ± 2° C; 12-hour light-dark cycle, lights on at 6:00 AM), and fed *ad libitum* Teklad chow (2018, 18% of calories from fat, 58% from carbohydrates, and 24% from protein. Envigo, Madison, WI, USA). All experimental procedures were approved by the Institutional Animal Care and Use Committee at Texas A&M University.

Experimental Design

Mice were switched to a semi-purified regular diet (D12450J, based on the AIN-93M formula, Research Diets Inc.) 2 weeks prior to the start of the experiment, and maintained on this diet until termination. After the diet washout period, animals received normal drinking water (control group) or DSS (2%) (MP Biomedicals; 36–50 kDa) in drinking water for 6 days, then switched back to normal drinking water; water was refreshed every 2–3 days. All mice were assessed for body weight, fecal consistency, and macroscopic fecal blood scores as described (1), with some modifications. Briefly, fecal consistency was scored as 0: normal stool, 1: soft but formed pellet, 2: very soft pellet, 3: diarrhea (no pellet), or 4: dysenteric diarrhea (blood in diarrhea). Rectal bleeding was scored as 0: no bleeding, 2: presence of visible blood in stool (red/dark pellet), and 4: gross macroscopic bleeding (blood around the anus). The disease activity score is calculated as the sum of fecal consistency and rectal bleeding scores; thus “0” denotes normal and “8” is the maximal disease activity score.

In Experiment 1, all mice (n = 10, 12, 9, and 8 for male-control, male-DSS, female control, and female-DSS groups, respectively) were subjected to conditioned fear training on Day 15, then context and cued fear recall tests on Day 16 (see below for detailed procedures). Animals were terminated on Day 17 after final scoring, brains were dissected and cut into 2 hemispheres along the midline, the right hemisphere was fixed in 4% paraformaldehyde overnight at 4° C then cryoprotected in 30% sucrose and kept at 4° C until further processing. The left hemisphere was further micro-dissected, hippocampus and cortex were collected, flash frozen, and stored at −80° C until further processing.

In Experiment 2, male C57BL/6J mice were subjected to control or DSS-induced colitis as described above, and brain tissues were collected on days 10 and 42 as described above (n = 4 for each group).

Pavlovian Conditioned Fear Test

Freezing behavior in a conditioned fear paradigm was measured as we described previously, with slight modifications (2). The test chamber (Habitest Modular System, Coulbourn Instruments) has clear Plexiglas sides and a grid floor bottom that is used to deliver a mild foot shock. The chamber is placed inside a sound-attenuation chamber. On the training day, mice were placed into the test chamber and allowed to explore for 2 min. The conditioned stimulus (CS, a 67 dB, 4 Hz tone) was presented for 30 s and followed immediately by a mild foot shock (2s, 0.3 mA) that served as the unconditioned stimulus (US). Two minutes later, a second CS-US pairing was presented. The FreezeFrame2 monitor system (Actimetrics) was used to control the timing of CS and US presentations and measure freezing behavior.

Mice were tested for contextual and cued fear memory tests 24 h after conditioning. For the contextual fear test, mice were placed back into the original test chamber for 5 min and freezing behavior was recorded. Three hours later, mice were tested for responses to the auditory CS in a new environment. For the CS test, a round red Plexiglas container with a grey smooth plastic bottom was inserted into the test chamber to alter the shape, texture, and color of the chamber. Vanilla extract was placed on metal panels under the light to alter the odor. Transfer cages were altered (no bedding). Mice were placed into this new chamber and freezing was recorded for 3 min during this ‘pre-CS’ phase. The auditory CS was then presented for another 3 min and freezing was recorded. Data for the auditory cued test were calculated as the percent freezing during the CS *minus* percent freezing in the pre-CS phase. In the present study, some male mice did not respond to the CS (cued response below the 5% threshold) and were excluded from data analysis. All female mice showed cued response above the 5% threshold.

Immunofluorescence Staining

Brains were embedded in OCT and serially sectioned in the sagittal plane into 40 µm thick sections using a Leica Cryostat (Leica Microsystems, Bannockburn, IL); sections were collected into 5 sets and stored in 0.1% sodium azide/PBS at 4°C until processing. Three brains per group collected on D17 were sectioned, and three sections per mouse brain were stained. Free-floating sections were washed with PBS/0.01% Triton X-100 (PBST) and permeabilized with 0.2% Triton X-100 in PBS at room temperature for 20 minutes, then blocked for one hour with 3% normal goat serum in PBST at room temperature. Sections were then incubated with chicken anti-GFAP antibodies (AB5541, Sigma) diluted in PBST/1% normal goat serum/2% BSA and incubated at 4°C overnight. The next day, sections were washed with PBST and incubated with

Alexa 488-conjugated goat anti-chicken antibodies (1:500, Invitrogen) in PBST at room temperature for two hours. Following this incubation, sections were washed with PBST three times for 10 minutes each, then counterstained with DAPI and mounted with Vectashield (Vector Labs). Fluorescent images were taken with a Leica DMi8 confocal system with 20x/0.40 HC PL FLUOTAR objective, using LASX software. All images were taken with the same settings (laser intensities, gain, offset, and pinhole) to compare the staining signals and patterns.

Image Analysis

Images of GFAP staining (Alexa 488 fluorophores; excited with 488 nm lasers and scanned with 510/530 bandpass filter) were processed with ImageJ. To estimate the size of GFAP-positive cells, images were first converted to binary mode, and cells that appeared intact were manually selected using the "Freehand selection" tool and areas measured. A total of 50 and 55 GFAP-labeled cells were measured from brain sections of the control and DSS-treated mice (3 sections per brain, 3 brains per group), respectively.

Quantitative Polymerase Chain Reaction Analysis

Hippocampal tissues were homogenized in lysis buffer and RNA isolated using the GenElute™ Mammalian Total RNA isolation kit (Sigma), following the manufacturer's protocol. cDNA was synthesized using the iScript System (Bio-Rad). Quantitative PCR was performed using primer sets for the indicated transcripts and SsoAdvanced Universal SYBR Green master mix (Bio-Rad), on CFX-384 thermocycler (Bio-Rad). The following mixtures of two primers were used for the detection of cDNAs: for *18s* (used as internal control), 5′-AGCCTGCGGCTTAATTTGAC-3′ and 5′- CAACTAAGAACGGCCATGCA-3′; for *Nfkb*, 5′-

AACAAAATGCCCCACGGTTA-3′ and 5′-GGGACGATGCAATGGACTGT-3′; for *Trem2*, 5′-CTGGAACCGTCACCATCACTC-3′ and 5′-CGAAACTCGATGACTCCTCGG-3′; for *Gfap*, 5′-TCTATGAGGAGGAAGTTCGAGA-3′ and 5′-TGCAAACTTAGACCGATACCA-3′; for *Il1b*, 5′-TGTTCTTTGAAGTTGACGGACCC-3′ and 5′-TCATCTCGGAGCCTGTAGTGC-3′; for *S100a8*, 5′-AAATCACCATGCCCTCTACAAG-3′ and 5′-CCCACTTTTATCACCATCGCAA-3′; for *Bdnf*, 5′-GGGTCACAGCGGCAGATAAA-3′ and 5′-GCCTTTGGATACCGGGACTT-3′. The $2^{-\Delta\Delta CT}$ method was applied to normalize against housekeeping gene *18s* and quantify relative expression.

Data analysis

Results were presented as mean ± SD, except for disease activity scores which were presented as mean ± SEM for clarity. Data were analyzed using unpaired t-test, one-way ANOVA followed by Tukey's multiple comparisons test, or two-way ANOVA followed by Tukey's multiple comparisons tests as appropriate. $P < 0.05$ indicates a significant difference between groups. Statistical analysis was done with GraphPad Prism 9.4 (GraphPad Software).

1. DeLuca JA, Allred KF, Menon R, Riordan R, Weeks BR, Jayaraman A, et al. Bisphenol-A alters microbiota metabolites derived from aromatic amino acids and worsens disease activity during colitis. Exp Biol Med (Maywood). 2018;243(10):864-75.
2. Wu CS, Chen H, Sun H, Zhu J, Jew CP, Wager-Miller J, et al. GPR55, a G-protein coupled receptor for lysophosphatidylinositol, plays a role in motor coordination. PLoS One. 2013;8(4):e60314.
